# Novel Biochip Platform for Nucleic Acid Analysis

**DOI:** 10.3390/s120608100

**Published:** 2012-06-11

**Authors:** Salvatore Pernagallo, Giorgio Ventimiglia, Claudia Cavalluzzo, Enrico Alessi, Hugh Ilyine, Mark Bradley, Juan J. Diaz-Mochon

**Affiliations:** 1 DestiNA Genomics Ltd., West Mains Road, Edinburgh EH9 3JJ, UK; E-Mails: salvatore.pernagallo@destinagenomics.com (S.P.); claudia@destinagenomics.com (C.C.); hugh@destinagenomics.com (H.I.); 2 STMicroelectronics, ANALOG, MEMS and SENSORS Group, Healthcare BDU, Stradale Primosole 50, Catania 95121, Italy; E-Mail: enrico.alessi@st.com; 3 School of Chemistry, University of Edinburgh, West Mains Road, Edinburgh EH9 3JJ, UK; E-Mail: mark.bradley@ed.ac.uk

**Keywords:** Lab-on-Chip (LoC), microarray, nucleic acid, dynamic chemistry, polymerase chain reaction (PCR), peptide nucleic acid (PNA), microfluidic, single nucleotide polymorphism (SNP), microRNA-122 (miRNA122), mengo virus (MGV), nucleic acid test (NAT), point-of-care (POC)

## Abstract

This manuscript describes the use of a novel biochip platform for the rapid analysis/identification of nucleic acids, including DNA and microRNAs, with very high specificity. This approach combines a unique *dynamic chemistry* approach for nucleic acid testing and analysis developed by DestiNA Genomics with the STMicroelectronics In-Check platform, which comprises two microfluidic optimized and independent PCR reaction chambers, and a sequential microarray area for nucleic acid capture and identification by fluorescence. With its compact bench-top “footprint” requiring only a single technician to operate, the biochip system promises to transform and expand routine clinical diagnostic testing and screening for genetic diseases, cancers, drug toxicology and heart disease, as well as employment in the emerging companion diagnostics market.

## 1. Introduction

The development of rapid diagnostic device platforms is one of the major scientific aims in the world of life-science research, drug discovery, medical diagnostics and biotechnology [1–7]. Several point-of-care (POC) diagnostic platforms have been launched in the past few years with some approved by the Food and Drug Administration (FDA) for *in vitro* diagnostic (IVD) applications [8–10]. Emerging nucleic acid test (NAT) technologies have allowed the development of applications for genotyping [single nucleotide polymorphisms (SNPs) and indel identification], epigenetic studies, array comparative genome hybridisation (aCGH), pre-natal screening, and microRNAs (miRNAs) profiling to name just a few, providing substantial future growth opportunities for LoC devices [11–18]. LoC approaches could overcome the technical limitations of nucleic acid mass screening by providing rapid, cheap and multiplexed assays [13,15].

**Figure 1.**
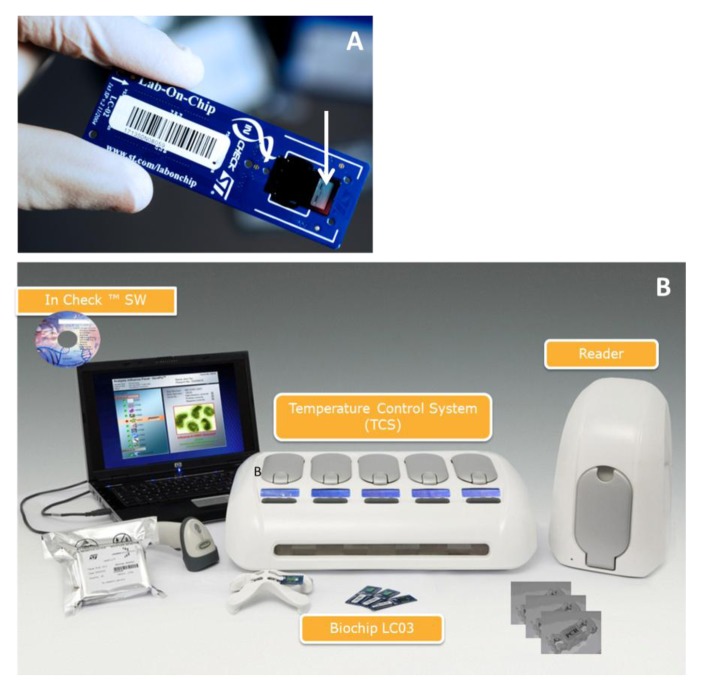
The main components of In-Check platform: (**A**) The Lab-on-Chip core device amplifies clinically relevant DNA samples by Polymerase Chain Reaction (PCR) and has an integrated custom low-density microarray (showed by the white arrow). (**B**) In-Check platform instruments. The Lab-on-Chip interfaces to the Temperature Control System (TCS) that actuates, monitors, and controls the parameters of the reaction. The TCS unit comprises five control modules with independent thermal protocols and random access capability. Optical signal acquisition is performed on a dedicated portable reader and processed by ST's specialized bioinformatics software. The software package allows users to easily monitor and control reaction processes, analyse the results and automatically generate diagnostic reports.

Among the advanced biochip-based technologies, STMicroelectronics has developed a disposable silicon-based micro electro mechanical system (MEMS) LoC device as a part of their “In-Check” platform [19–21]. This platform combines all the functions needed to identify given oligonucleotide sequences in a sample and includes microfluidic handling, a miniaturized PCR reactorand a nucleic acid microarray detection module (Figure 1).

The In-Check platform has already been used successfully to amplify human genome sequences and detect human genome mutations, such as the gene associated with β-thalassemia as well as the detection of viral infectious diseases with full integration of the PCR amplification with subsequent microarray detection [22–24].

Previously, the chemical-based approach for nucleic acid testing (Chem-NAT) commercialised by DestiNA Genomics had been validated by genotyping, with 100% read accuracy, using DNA from mouth swabs from Cystic Fibrosis (CF) patients and mass spectrometry (MALDI-ToF) for analysis [25].

**Figure 2.**
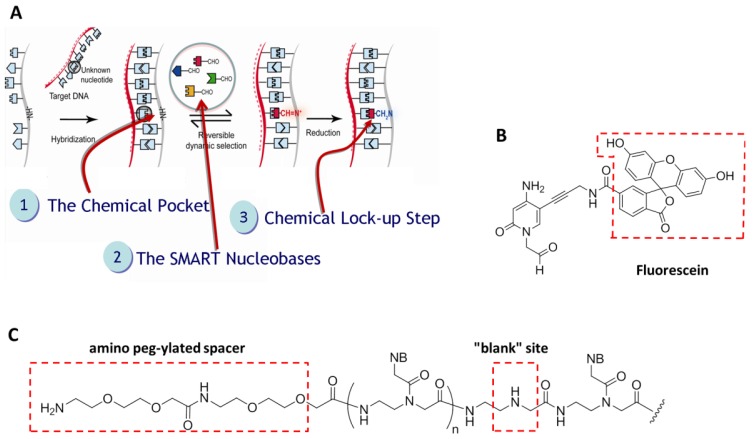
(**A**) The steps involved in DestiNA Genomics chemical-based approach for nucleic acid testing (Chem-NAT). (Copyright Wiley-VCH Verlag GmbH & Co. KGaA, Reproduced with permission) [26]. DestiNA probe with the target sequence to be detected creates a molecular pocket (step 1) that allows the specific incorporation of a DestiNA SMART nucleobase (step 2) which is then chemically locked into position (step 3). SMART nucleobase incorporation can be directly detected by Mass Spectrometry. (**B**) Structure of the fluorescein labelled aldehyde Cytosine (FITC-C_CHO_) for detection using fluorescence reporter system. (**C**) Structures of a DestiNA probe containing a N-terminated amino peg-ylated spacer for being covalently immobilised onto surfaces.

Briefly, DestiNA core technology takes advantage of dynamic chemistry for nucleic acid sequence specific recognition using aldehyde-modified natural nucleobases (so called SMART nucleobases), and probes based on peptide nucleic acid (PNA), containing an “abasic” position (DestiNA probes) which can be made complementary to any target nucleic acid sequence (Figure 2(A)) [26].

A major feature of Chem-NAT is that false positives are difficult if not impossible to create as nucleobase incorporation can only occur in the presence of target templating nucleic acid strands Figure 2(A).

While mass spectrometry allows single base discrimination and multiplexing capabilities due to molecular weight differences between SMART nucleobases, fluorescence based assays require the DestiNA SMART nucleobases to be fluorescently-labelled and the DestiNA probes to be modified to allow their covalent immobilisation on surfaces Figure 2(B,C).

Multiplexing can be achieved by printing probes at defined XY coordinates and by incorporation of the correct fluorescently-labelled SMART nucleobase into the chemical pocket following duplex hybridisation. Such an application allows the use of label-free nucleic acids.

Herein, a proof-of-concept study which integrates DestiNA Genomics Chem-NAT with STMicroelectronics In-Check LoC platform is described, delivering a novel biochip platform for the rapid detection of nucleic acids with high sensitivity and specificity.

The novel biochip platform was evaluated and validated for detection of synthetic small RNAs (sRNAs) based on microRNA-122 (miRNA122) and mengo virus RNA (MGV). This biological model represents the first steps in the development of a novel suite of assays for the medical diagnostic field. Integration of DestiNA technology with the STMicroelectronics In-Check LoC creates a highly innovative product with a true diagnostic potential and utility, for rapid detection of nucleic acids with benefits in terms of result consistency, time, cost, and ease of use.

## 2. Experimental Procedure and Methods

### 2.1. General

STMicroelectronics In-Check LoC platforms were fabricated as described previously [19]. Commercially available reagents and buffer for the functionalization of the LoC surfaces were used without further purification. Hydrogen peroxide (29%), ammonium hydroxide (25%), hydrochloridric acid (37%) and methanol were purchased from Sigma Aldrich (Poole,UK) and were used as received. Spot buffer (Nexterion Spot) and hybridisation solution (Nexterion Hyb) were purchased from SCHOTT and were used as received. All synthetic RNA oligomers were purchased in desalted form from Microsynth AG (Balgach, Switzerland).

### 2.2. Instrumentations

A microdrop inject printer equipped with a micro-pipette (AD-K-501 70 μm diameter nozzle) (Microdrop Technologies GmbH, Muehlenweg, Norderstedt, Germany) and a BioAnalyzer 4F/4S equipped with a light scanner (LaVision BioTech GmbH, Bielefeld, Germany) were used. DestiNA probe aqueous solutions concentrations were determined using an Agilent 8453 spectrophotometer. Hybridisation was carried out on a Q-Hyb10 Hybridisation Oven Incubation System Deltaspin (Quanta Biotech Ltd., Surrey, UK).

### 2.3. Probe Synthesis and Purification

DestiNA probes terminate with an amino PEG group and were prepared by solid-phase synthesis (SPS) using Fmoc/Bhoc protected monomers as described previously [25] and were characterized by MALDI-TOF mass spectrometry (see Figures S1 and S2 in the Supplementary Information).

### 2.4. Synthesis of FITC Labelled Aldehyde Cytosine

Aldehyde-modified cytosine, tagged with a fluorescein molecule was prepared following the synthetic route described elsewhere [27] and characterized by MALDI-TOF mass spectrometry (see Figure S3 in the Supporting Information).

### 2.5. Probes Immobilization

Attachment of DestiNA probes was carried out according to manufacturer's protocols (SCHOTT). Briefly, amino peg-ylated spacer probes were dissolved in 1X spotting solution to give 100 μM solutions. The solution was spotted onto the epoxysilane-functionalised LoC, followed by incubation under humidity for 30 min in a hybridisation chamber box (Genetix). Thereafter, the LoCs were washed for 5 min in 0.1% Triton X-100, 2 × 2 min in 1 mM HCl, 10 min in 100 mM KCl and 1 min in deionized water. After washing, the LoCs were blocked in 150 mM phosphate buffer containing 50 mM dimethylamine, pH 9, for 15 min at 50 °C with stirring. The LoCs were washed for one min with deionized water, dried in an oil-free nitrogen stream and stored at room temperature until use.

### 2.6. Validation of DestiNA Probes Immobilization

Features on the LoCs were hybridized with complementary labelled synthetic sRNAs (see Table S1, target 1 and 2, in the Supporting Information). In short, in a 0.2 mL Eppendorf, 10 μL of 100 μM of labelled synthetic sRNAs were dissolved in 20 μL of diH_2_O. Samples were mixed with 30 μL of hybridisdation buffer and in accordance with the guidelines provided by STMicrelectronics, this solution was loaded into LoCs (through to the two inlets to reach the microarray area). The arrays were covered with two accessories in plastic (clamps) for array sealing and inserted into the Hybridisation Oven for hybridisation. Hybridisation was standardised by treatment at 50 °C for 2 h. Thereafter, clamps were removed, and the LoCs were washed for 10 min each in 2 × SSC + 0.1% SDS, 2 × SSC and then 0.2 × SSC (all wash steps were performed at room temperature). LoCs were dried using an oil-free nitrogen stream and scanned using a 100 ms exposure time using emission and detection filters appropriate for FITC.

### 2.7. DestiNA Reaction on LoC

A 0.2 mL Eppendorf containing 5 μL of a 100 μM unlabelled miRNA122 oligomer aqueous solution (see Table S1, target 3 in the Supporting Information) was placed in a heater (Techne TC-312 Thermocycler) at 95 °C for 5 min, then cooled to 40 °C before being combined with 10 μL of NaBH_3_CN (1 M) and 15 μL of FITC labelled aldehyde cytosine (FITC-C_CHO_) (100 μM in 0.5% DMSO aqueous solution). Samples were mixed with 30 μL of hybridisation buffer (final volume 60 μL) and loaded onto the LoC as described above. The arrays were covered with the two clamps and placed into the hybridisation oven at 50 °C for 2 h. After the reaction the LoCs were washed, dried and scanned as described above.

## 3. Results and Discussion

In a first phase of this feasibility study, a protocol to print DestiNA probes onto LoC using an inkjet printer was developed. Once the printing and blocking conditions were optimised, a second phase was carried out in order to check if DestiNA probes, covalently immobilized on the LoC, were able to form duplexes with complementary synthetic sRNA oligomers. In a final stage dynamic incorporation of FITC-C_CHO_ was tested to determine if high specificity was achieved on the novel biochip platform.

### 3.1. DestiNA Probes for In-Check

In this study two different RNA strands mimicking natural sRNAs were used to validate the methodologies. The two objects of this study were related to miRNA122 and MGV. miRNA122 is a 22 nucleotide long single strand RNA found in high concentrations in human plasma of patients who have overdosed on paracetamol, becoming a prospective biomarker of liver damage [28]. MGV can be passed to humans through food intake, mainly in shellfish and analysis in the food chain is important to avoid major outbreaks of hepatitis A [29]. The MGV transcript is over 3,000 Kb long and the target region selected was chosen based on recommendation of the European Committee for Standardisation (CEN/TC 275/WG6/TAG4-viruses in foods).

**Figure 3.**
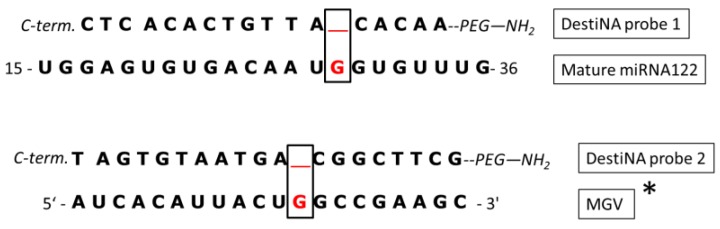
miRNA122 and MGV were interrogated using two different DestiNA probes (1 and 2) 18 and 20 mers respectively. Incorporation of FITC-C_CHO_ provides proof-reading, indicating the presence of the complementary oligomers. Only with perfect complementarity the FITC-C_CHO_ SMART nucleobase will be incorporated. Probe design was carried out using publicly available database (miRBase Accession Number: MIMAT0000421). MGV was designed using the real-time probe Mengo147 previously published [29]. “*” The single-stranded DNA probe Mengo147 was an identical version of the mengo virus genomic RNA except that “T” bases were replaced with “U” bases.

To investigate and discriminate between the chosen targets, two different DestiNA probes, one to clamp the mature miRNA122 sequence and the one targeting MGV genomic RNA, were designed and synthesised. The design aligned the N-terminal end of the probes with the 3′ end of their target having the “blank” position lined up opposite to a guanidine residue under interrogation, as shown in Figure 3.

DestiNA probes were reacted chemoselectivity with the epoxy groups that coat the silicon-based microarray surface of the In-Check LoC.

**Figure 4.**
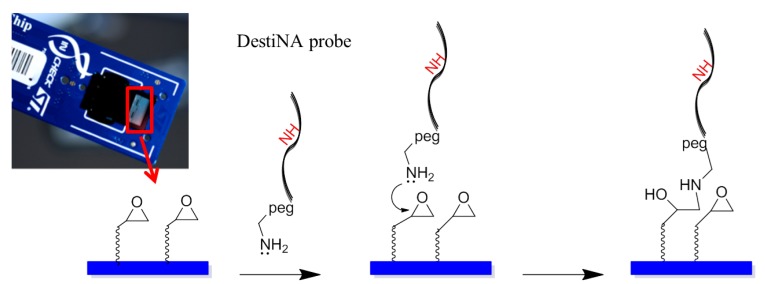
Modified DestiNA probes immobilization. DestiNA probes were covalently attached through an epoxide ring-opening reaction *via* their primary amines.

Figure 4 shows an amino-modified DestiNA probe reacting with the epoxysilane surface of the LoC (a reaction that does not need additional baking or UV cross-linking steps). Molecular spacers (PEG group) between the probes and the LoC epoxy groups facilitate interactions between the printed DestiNA probes and their target binding partners in solution.

## 3.2. Printing and Blocking Optimization

**Figure 5.**
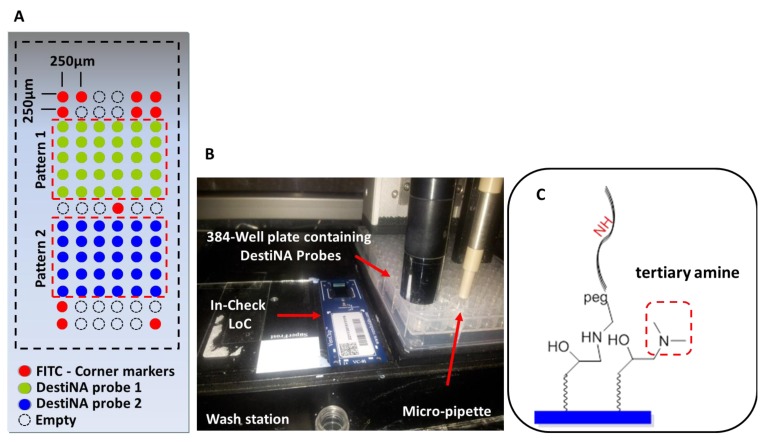
(**A**) Graphic layout of the microarray. (**B**) Printing station with a microdrop printer GmbH (Norderstedt, Germany) containing a micro-pipette with a 70 μm diameter nozzle. (**C**) Efficient blocking using 150 mM phosphate buffer containing 50 mM dimethylamine. Red square shows the tertiary amines formed.

An important stage in the construction of the novel LoCs was defining the microarray probe layout. The two test probes were printed onto two symmetrical portions of the array (pattern 1 and 2) and arranged in 15 rows and six columns (90 features in total). Taking into consideration the size of the microarray of 3.7 mm × 1.45 mm, it was decided to create a spot pitch of 250 μm Figure 5(A). Corner control FITC-labelled DNA oligomers (see Table S1 in the Supporting Information) were printed to identify array orientation. Empty positions to evaluate background noise were provided. Overall, 32 positions were allocated to the control probes and 64 to the specific probes Figure 5(A).

Printing was performed using a proprietary non-contact inkjet printing process in which DestiNA probes were deposited uniformly onto the LoC microarray area. The precise inkjet process enabled the delivery of extremely small, accurate volumes (picoliters) of the chemicals Figure 5(B).

Efficient blocking of reactive surface groups after arraying was critical for a reduced fluorescence background. The LoC was blocked with a 150 mM phosphate buffer containing 50 mM dimethylamine. The tertiary amines engendered prevented reactions with the aldehyde groups of the FITC-C_CHO_ Figure 5(C). Standard SCHOTT blocking buffer (containing ethanolamine) blocking approach were excluded as this would give rise to secondary amines on the surface which could react with the aldehyde groups of the fluorescently-labelled SMART nucleobase.

## 3.3. Printing Validation with Synthetic Oligonucleotides

**Figure 6.**
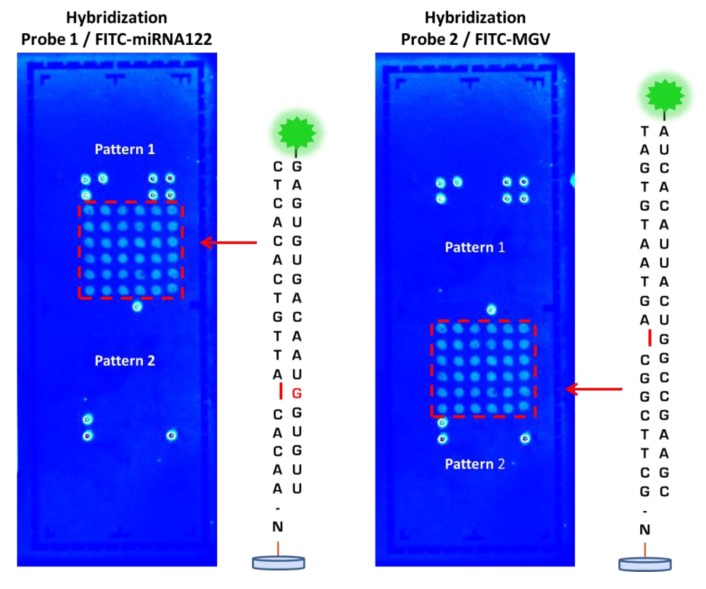
LoCs scanned after the hybridisation of DestiNA probes with either synthetic miRNA122-FITC (**left**) or MGV-FITC (**right**). LoCs were scanned using a 200 ms exposure time using emission and detection filters appropriate for FITC.

Following printing of the probes on the LoC microarray area, the performance of the DestiNA probes was initially checked by hybridizing complementary fluorescently-labelled sRNA oligomers (see Table S1, target 1 and 2 in the Supporting Information). Figure 6 shows fluorescence scanning images of those areas, clearly demonstrating the ability of the immobilised DestiNA probes to recognise and form duplexes with complementary nucleic acid strands.

Good printing performance and excellent spot morphology for both probe 1 and 2 was observed. Pattern 1 (left image) shows where the oligo miRNA122 FITC plus complementary DestiNA probe 1, attached to the LoC surface, has correctly hybridised and fluoresces. Pattern 2 (left image) shows no fluorescence, as the oligo miRNA122 FITC had no complementarity to DestiNA probe 2 and, was washed away. Additionally, Pattern 2 (right image) shows where the oligo MGV FITC plus complementary DestiNA probe 2 has correctly hybridised and fluoresces, while Pattern 1 (right image) shows no fluorescence, as the oligo MGV had no complementarity to DestiNA probes 1.

### 3.4. DestiNA Reaction on In-Check LoC Platform

**Figure 7.**
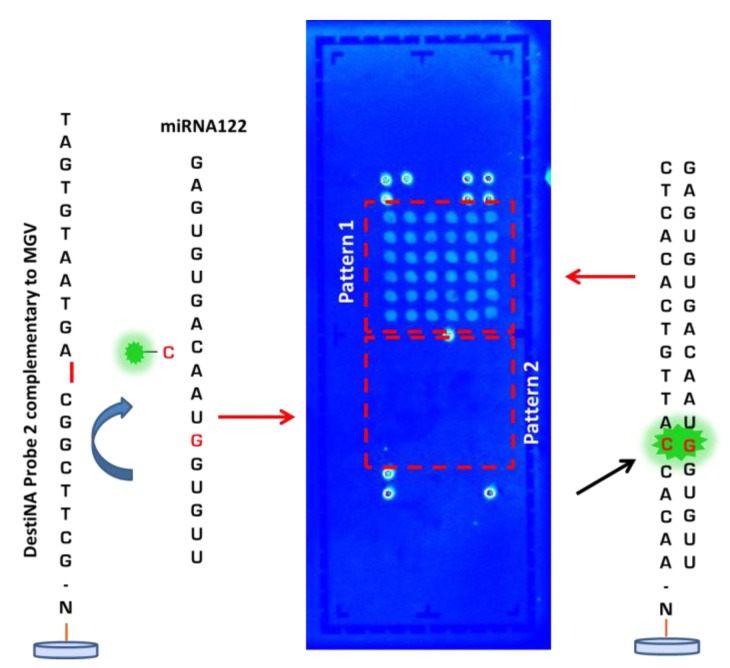
LoC scanned after DestiNA reaction with FITC-C_CHO_. Red square (pattern 1) shows where the oligo miRNA122 plus complementary DestiNA probe 1 attached to the array has correctly hybridised and fluorescence due to FITC-C_CHO_ incorporation (showed by black arrow). On the other hand, red square (pattern 2) does not show fluorescence due to an absence of hybridisation between oligo miRNA122 plus DestiNA probe 2 (FITC-C_CHO_ incorporation not take place).

After optimising the printing, blocking conditions and validating the capability of DestiNA probes to efficiently hybridise only with complementary sequence sRNA oligomers, the ability to achieve a DestiNA Chem-NAT dynamic chemistry protocol on the LoC devices was explored. Unlabelled miRNA122 (see Table S1, target 3 in the Supporting Information) was used as template strand and FITC-C_CHO_ nucleobase was used with LoCs previously arrayed with DestiNA probes (Figure 5(A)). The resulting LoC scanning (Figure 7) showed the ability of the DestiNA technology to identify miRNA122 oligomer on In-Check, using a highly selective incorporation of the fluorescently-labelled cytosine.

Background was at a very low level, demonstrating excellent blocking and the benefit of the proof reading step using the DestiNA SMART base protocol.

Selective incorporation of FITC-C_CHO_ into the PNA/RNA hybrid through dynamic chemical reactions can take place ONLY when miRNA122 forms a perfect duplex with the designed DestiNA probes (pattern 1). Pattern 2 shows no fluorescence because non-complementary (miRNA122) has not been hybridised with the PNA probe 2. This demonstrated the selective incorporation of the “correct” base when the target nucleic acid forms a perfect duplex. The result confirms that the DestiNA fluorescence detection approach to nucleic acid discrimination reported here could be ported into the LoC platform, facilitating the analysis of label-free nucleic acid. The result further indicates that the approach used with miRNA122 herein could be extended to more generic, direct nucleic acid analysis such as allele-discrimination.

## 4. Conclusions

In this manuscript we have given an overview of a “Proof of Concept” study for testing the integration of DestiNA Genomics and STMicroelectronics technologies. All the key proof of concept objectives were achieved, including effective DestiNA probe printing and DestiNA reactions, demonstrating successful translation of DestiNA reagents onto the In Check LoC platform. This study represents a first step in merging the two technologies to create a suite of novel medical diagnostic assays capable of delivering a novel biochip platform for the rapid detection of nucleic acids with high sensitivity and specificity.By combining the novel chemical-based methods with the Lab-on-Chip platform, clinically valuable innovation is achieved through:

1. The unique high affinity and selective hybridisation between DestiNA probes and target DNA or RNA, superior to standard DNA/DNA or DNA/RNA hybridisations.
2. The innovative DestiNA probes, that contain a “blank”/nucleobase free position within the probe, enable a unique proof-reading step to occur. A rapid and selective incorporation of complementary fluorescently labelled SMART nucleobase into the PNA/RNA hybrid can ONLY occur if the target nucleic acid forms a perfect duplex with the designed DestiNA probe.
3. Flexibility to design “personalized” and low cost multiplex assays for target detection in individuals able to be undertaken by laboratory technicians.

The integration and combination of DestiNA Genomics and STMicroelectronics technologies are very promising and potentially suitable for developing a highly innovative next generation system capable of true diagnostic value and utility for the rapid detection of nucleic acids. Benefits for health care providers will be cost benefits in term of reliability, time, and ease of use.

## Supplementary Material
