# Supplementary material for: Novel Biochip Platform for Nucleic Acid Analysis
Source: Sensors (Basel). 2012 Jun 11;12(6):8100–11. doi: 10.3390/s120608100 (PMC3436018; doi:10.3390/s120608100)
Supplement: Supplementary file 1 [file sensors-12-08100-s001.pdf]

OPEN ACCESS
sensors
ISSN 1424-8220
www.mdpi.com/journal/sensors

Supplementary Information

Novel Biochip Platform for Nucleic Acid Analysis. *Sensors* 2012, *12*, 8100-8111

Salvatore Pernagallo [1,†], Giorgio Ventimiglia [2,*,†], Claudia Cavalluzzo [1], Enrico Alessi [2], Hugh Ilyine [1], Mark Bradley [3] and Juan J. Diaz-Mochon [1,*]

[1] DestiNA Genomics Ltd, West Mains Road, Edinburgh EH9 3JJ, UK; E-Mails: salvatore.pernagallo@destinagenomics.com (S.P.); claudia@destinagenomics.com (C.C.); hugh@destinagenomics.com (H.I.)
[2] STMicroelectronics, ANALOG. MEMS and SENSORS Group, Healthcare BDU, Stradale Primosole 50, Catania 95121, Italy; E-Mail: enrico.alessi@st.com (E.A.)
[3] School of Chemistry, University of Edinburgh, West Mains Road, Edinburgh EH9 3JJ, UK; E-Mail: mark.bradley@ed.ac.uk (M.B.)

† These authors contributed equally to this work.

* Authors to whom correspondence should be addressed; E-Mail: giorgio.ventimiglia@st.com (G.V.); juan@destinagenomics.com (J.J.D.M.); Tel.: +39-095-740-4344 (G.V.); +44-0-131-650-4821 (J.J.D.M.).

---

1. Figure S1. MALDI-TOF mass spectrometry of DestiNA probe 1.
2. Figure S2. MALDI-TOF mass spectrometry of DestiNA probe 2.
3. Figure S3. MALDI-TOF mass spectrometry of fluorescein-labelled cytosine.
4. Table S1. Sequences of synthetic RNA/DNA oligomers.

MALDI-TOF Spectra

Figure S1. MADLI-TOF MS of Probe 1: Amino-peg- AAC AC_ ATT GTC ACA CTC. Exact Mass: 4,920.04, Molecular Weight: 4,922.79.

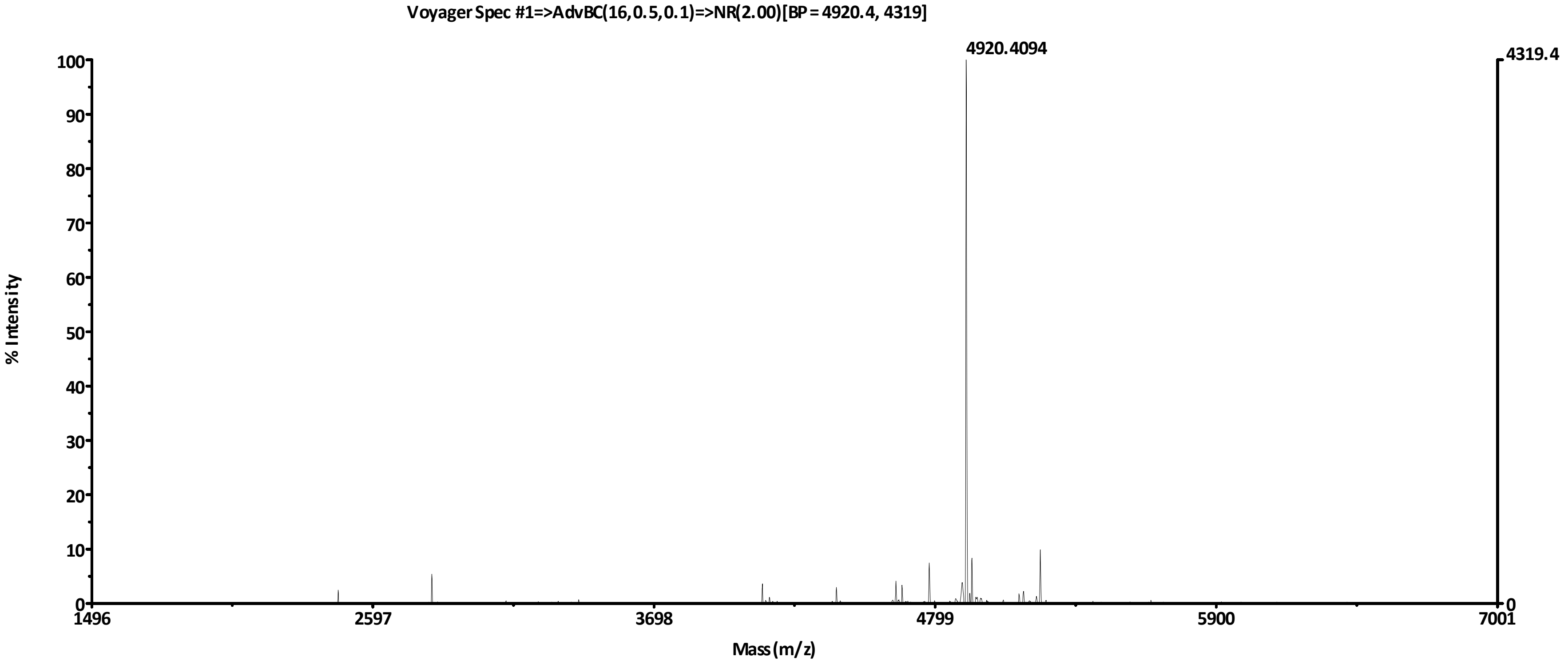

Figure S2. MADLI-TOF MS of Probe 2: Amino-peg- GC TTC GGC _AG TAA TGT GAT. Exact Mass: 5,564.25, Molecular Weight: 5,567.35.

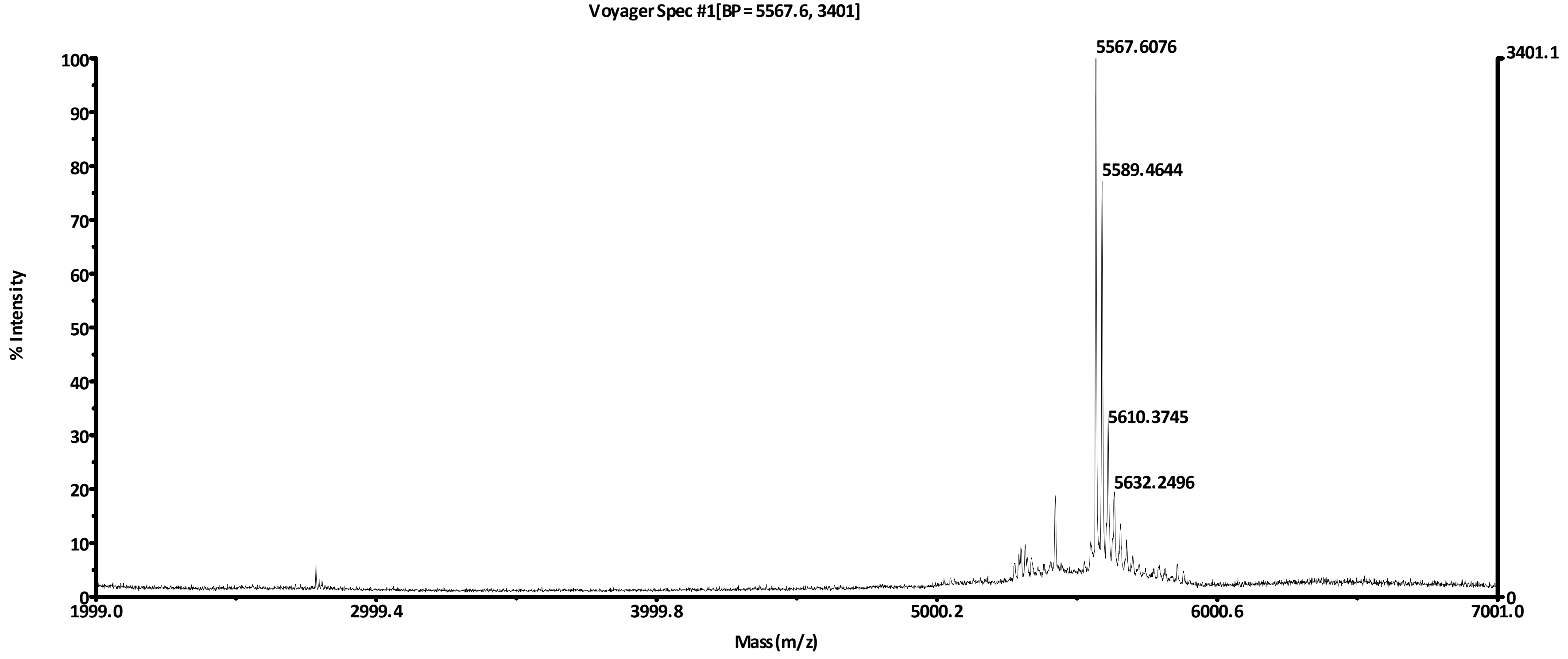

Figure S3. MADLI-TOF MS of fluorescein-labelled cytosine. Exact Mass: 563.13, Molecular Weight: 563.41.

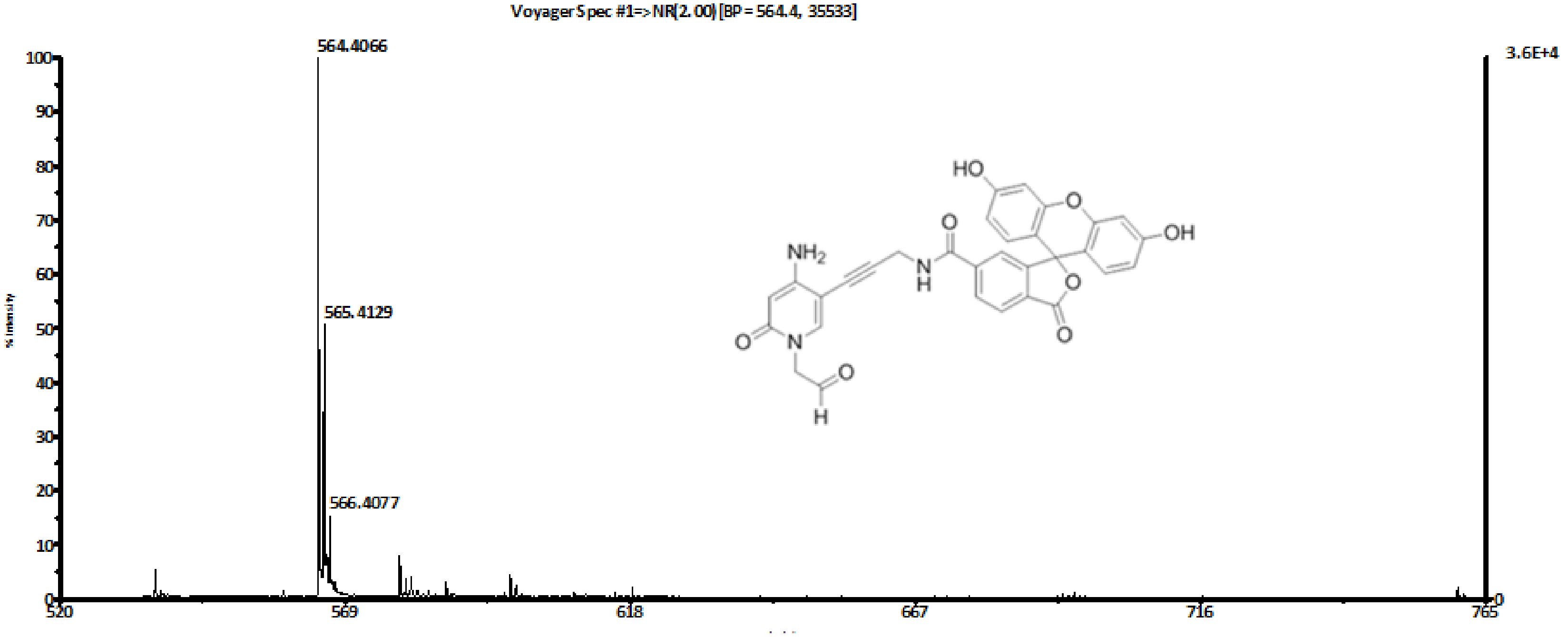

Table S1. Sequences of synthetic RNA/DNA oligomers.

Target	Name	Sequence (5'→3')	5' Modif.	3' Modif.
1	miRNA122	GAGUGUGACAAUGGUGUU	FITC	NONE
2	MGV	AUCACAUUACUGGCCGAAGC	FITC	NONE
3	miRNA122	GAGUGUGACAAUGGUGUU	NONE	NONE
Corner Marker		ACTATCATCTAAACT	Amino C6	FITC

© 2012 by the authors; licensee MDPI, Basel, Switzerland. This article is an open access article distributed under the terms and conditions of the Creative Commons Attribution license (http://creativecommons.org/licenses/by/3.0/).
